# Qualitative insights into mental health treatment through telemedicine during the COVID-19 crisis: a natural experiment in community mental health centers

**DOI:** 10.1186/s40352-024-00282-9

**Published:** 2024-07-20

**Authors:** Brittany J. Hood

**Affiliations:** Texas A&M International University, 5201 University Boulevard, Academic Innovation Center (AIC) 314, Laredo, Texas 78041 United States

**Keywords:** Criminal justice, Serious mental illness (SMI), Telemedicine, Community mental health centers, Mental health, Treatment, Childcare, Transportation, Qualitative, Confidentiality

## Abstract

**Background:**

The COVID-19 pandemic exacerbated existing mental health challenges and introduced new ones, particularly among vulnerable populations such as individuals within the criminal justice system, who disproportionately experienced employment, financial, and housing issues. As mandatory lockdowns and social distancing mandates were implemented, the United States saw unprecedented interruptions to treatment. Telemedicine emerged as a transformative tool in alleviating new and existing treatment barriers. Yet, limited empirical research has examined the impact and implications of telemedicine on mental health treatment in criminal justice populations.

**Methods:**

The timing of this study’s data collection overlapped with the spread of COVID-19 in the United States and provided a unique opportunity to examine the impact of telemedicine as part of a natural experiment. Utilizing interviews with 61 community mental health center service providers, this study qualitatively examined service providers’ experiences in treating criminal justice-involved individuals with serious mental illness who were receiving mental health treatment through telemedicine.

**Results:**

Service providers expressed satisfaction with telemedicine in addressing client transportation and childcare barriers while increasing engagement. Service providers voiced new concerns regarding clients’ confidentiality, digital literacy, and limitations to gathering non-verbal client information during virtual treatment.

**Conclusions:**

Mental health treatment offered through telemedicine mitigates barriers to treatment that disproportionately affect criminal justice clients. Despite its benefits, challenges like access to reliable internet and to internet-enabled devices, confidentiality concerns, and information gathering must be addressed to achieve optimal and equitable mental health treatment through telemedicine. The findings support the continued use of telemedicine in mental health treatment delivery for this population.

## Background

The COVID-19 pandemic underscored the profound impact that Disasters and economic downturns have long been known to impact mental health significantly, often leading to heightened stress, depression, anxiety, and even severe outcomes such as suicide and drug overdoses (Beaglehole et al., 2018; Norris et al., 2002a; Norris, Friedman, Watson, Norris et al., 2002a, b). Factors such as social isolation, economic instability, and uncertainty furtherexacerbated these challenges (Gonzalez et al., 2021; Hossain et al., 2020; Lee et al., 2021). Individuals within the criminal justice system are particularly vulnerable, facing higher rates of unemployment, food and housing insecurity, and disruptions to essential services like education and childcare (Schwalbe & Koetzle, 2021).

In response to these challenges, criminal justice agencies have increasingly adopted technological solutions. Virtual courtroom proceedings, implementing changes to policing practices, switching to the remote supervision of individuals on probation or parole, and initiating telemedicine services within correctional facilities for mental health care delivery and remote medication management for individuals with mental illness have become more prevalent (Baldwin et al., 2020; Buchanan et al., 2020; Jennings & Perez, 2020; Marcum, 2020; Schwalbe & Koetzle, 2021; Vera Institute of Justice, 2020; Viglione et al., 2020). These technologies have addressed several longstanding barriers, such as transportation issues, and have facilitated the adjudication and supervision of criminal justice-involved individuals (Baldwin et al., 2020; Burton et al., 2021; Oluyede et al., 2022; Viglione et al., 2020).

Despite these advancements, the delivery of healthcare, particularly mental health treatment, has faced disruptions, leading to delays and interruptions (Gonzalez et al., 2021; Lee & Singh, 2021; Murphy et al., 2021). Telemedicine has emerged as a critical alternative to face-to-face treatment, expanding access to care and offering unprecedented flexibility (Koonin et al., 2020). This modality has helped alleviate barriers such as limited insurance coverage, stigma associated with mental illness, and socioeconomic disparities (Gary, 2005; Leong & Kalibatseva, 2011; Misra et al., 2021; Pabayo et al., 2022).

Telemedicine has shown promise in reducing barriers to care for various populations, including parents with childcare responsibilities and individuals in remote or underserved areas as telemedicine bridged geographical gaps, enabling access to care even across state lines (Katzow et al., 2020; U.S. Department of Health and Human Services, 2023). Research indicates a significant increase in service utilization, particularly addressing opioid use disorder treatment, highlighting the potential impact of telemedicine to expand access and improve outcomes (Schwalbe & Koetzle, 2021; Swann et al., 2022). The elimination or reduction of these barriers not only led to expanded access to treatment but also brings unexpected benefits, including expedited treatment initiation time and increased scheduling flexibility (Barney et al., 2020; Frank et al., 2021; Kruse & Heinemann 2022).

Despite the advantages of and enhanced flexibility associated with telemedicine, its adoption hinged upon access to the requisite technology, including to reliable internet connectivity and devices such as computers, tablets, or smartphones (Dir et al., 2022; Krider & Parker, 2021; Schwalbe & Koetzle, 2021; Zaller et al., 2023). Financial constraints, especially prevalent in underserved communities, pose significant challenges to maintaining and utilizing this technology (Song et al., 2021). Rural areas face additional barriers due to limited internet access (Ojha & Syed, 2020; Oluyede et al., 2022; Song et al., 2021). Digital literacy also plays a crutis another critical aspect shaping access, with some facing a steep learning curve in adopting the technology necessary for virtual adjudication, supervision, or treatment. Concerns about data privacy and trust, especially prevalent in the criminal justice population, are additional factors that might affect their willingness to adopt telemedicine (Zaller et al., 2023). Paradoxically, while telemedicine alleviated transportation-related barriers, it may have decreased treatment access for individuals with limited resources (Delisle-Reda et al., 2022).

Research from the service provider perspective offers valuable insights into the impact of technology and client barriers on mental health treatment. Although limited in scope, these studies shed light on both impediments and possible solutions. For example, Krider and Parker (2021) that service providers in rural areas were generally satisfied with virtual options in mental health and criminal justice settings, noting their practicality and ability to maintain client confidentiality. However, previous research suggests that criminal justice-involved individuals receiving treatment may have concerns about confidentiality and privacy (Batastini & Morgan, 2016).

Dir and colleagues (2022) conducted a comprehensive study on telemedicine, analyzing substance use treatment and criminal justice in a community setting. They found that while telemedicine was generally viewed positively, service providers express concern with long-term feasibility, particularly regarding the minimum number of required office visits. The need for further research on the outcomes and implications of telemedicine for criminal justice-involved populations, especially for those requiring behavioral health services, is emphasized.

Despite advancements in mental health treatment delivery, there remains a notable gap in research, with the most studies focusing on non-criminal justice populations (Blanco et al., 2021). This oversight fails to account for the unique challenges faced by individuals within the criminal justice system, particularly those with serious mental illnesses (SMI). Research on the impact of telemedicine on criminal justice-involved individuals is limited, mainly focused on those with substance use disorders (Delisle-Reda et al., 2022; Dir et al., 2022; Donelan et al., 2021; Swann et al., 2022). Moreover, the perspectives of service providers remain largely underexplored (Dir et al., 2022; Donelan et al., 2021; Krider & Parker, 2021).

This study aims to contribute to this line of inquiry using interviews with service providers in community mental health centers (CMHCs) across one Midwestern state. This study’s inception coincided with a significant shift in telemedicine adoption, providing a unique opportunity to investigate it’s impact on treatment access for individuals involved in the criminal justice system. This timing allowed for a secondary analysis of service provider’s discussions, observing changes in perceptions and practices regarding client barriers to treatment access. By leveraging this coincidental timing, the study evolved into a natural experiment, offering robust insights into the real-world implications of telemedicine adoption and underscoring the significance of this study’s findings in informing both policy and practice in these critical areas.

## Methods

This study builds upon a cross-sectional parent study consisting of 61 in-depth, semi-structured interviews with Indiana CMHC service providers who specialize in treating individuals with criminal justice involvement and SMI. The initial study primarily investigated the influence of organizational-level variables and legal constraints on treatment decisions made by CMHC service providers. Data collection occurred between December 2019 and April 2020, coinciding with the onset of the COVID-19 pandemic. Service providers were asked about (1) their education and relevant training, (2) their prior experiences in working with forensic clients, (3) the prevalence of each SMI diagnosis in their criminal justice-involved clients, and (4) how goals, policies, budget, and legislation could aid in or limit treatment decisions.

Although the study was designed prior to the pandemic and did not incorporate specific questions about COVID-19 for service providers, the timing presented a unique opportunity to investigate the impact of telemedicine on treatment access for individuals with SMI involved in the criminal justice system, employing a natural experimental framework. Natural experiments, which utilize experimental or non-experimental designs, allow researchers to observe naturally occurring phenomena beyond researchers’ control (Leatherdale, 2019). In this instance, the introduction of telemedicine served as such a phenomenon, facilitating a comparison of how service provider’s discussions regarding clients’ barriers changed from before and during the implementation of telemedicine amidst the pandemic. Findings derived from this study design allow for robust conclusions to be drawn regarding the implication of telemedicine on accessing treatment for individuals with SMI involved in the criminal justice system.

### Recruitment

In the community, criminal justice-involved persons are most likely to receive treatment for SMI in CMHCs. Thus, recruitment targeted service providers in CMHCs across Indiana, comprising 24 independent organizations across the state and have a combined total of 45 offices. Administrators held titles including Clinical Supervisors, Directors of Clinical Services, Directors of Behavioral Health, or Coordinators.

Recruitment began by identifying an administrator at each organization through phone calls. The researcher then obtained contact information for each administrator. An introductory email was sent, outlining the study’s nature and purpose along with the IRB-approved study information sheet. Administrators who did not initially respond received monthly follow-up e-mails for four months during data collection. Only three administrators declined participation, accounting for 6.7% of the total sampling frame. Thirteen administrators (28.9%) did not respond, and an additional five administrators (11.1%) responded but did not provide necessary contact information for service provider recruitment. Non-participating CMHCs were predominantly rural; nevertheless, over 34% (*n* = 21) of service providers in the final sample operated in rural counties. Ultimately, sixteen administrators (35.6%) agreed to participate in the study.

Next, the administrator supplied contact information for any service providers directly involved with treating clients with SMI and criminal justice involvement. For this study, current criminal justice involvement was defined as “any individual who was recently arrested, on probation or parole, under the jurisdiction of a specialty court, a Department of Child Services referral, mandated by the court for treatment, house arrest, ankle monitor, or on work release.” Due to the absence of a comprehensive list of service providers meeting the eligibility criteria, random selection was not feasible, which caused the researcher to depend on administrator-provided contacts. Consequently, recruitment relied on administrator-provided contact information. Purposive (74%) and snowball (26%) sampling methods were then employed to recruit interviewees through email. Recruitment emails explained the study purpose, inviting interested service providers to respond to the researcher via e-mail or phone. Unfortunately, because some organizations chose to send the recruitment e-mail directly to their service providers rather than provide their contact information, it was not possible to determine how many service providers received recruitment e-mails. Interviews were conducted via Zoom, lasting one hour on average, and were recorded with verbal permission to be transcribed verbatim.

### Service providers and their caseloads

The sample was predominately non-Hispanic white (95.0%) females (74.0%), most of whom held master’s degrees (80.3%). Respondents were not specifically asked about their discipline(s) or area(s) of study. However, it is worth noting that many interviewees voluntarily provided this information. The most commonly mentioned degrees were Licensed Clinical Social Worker (LCSW) and Licensed Mental Health Counselor (LMHC), accounting for 16.0% and 8.0% of respondents, respectively.

Service providers were asked to estimate the percentage of their criminal justice clients who were diagnosed with SMI. It is important to note that individuals can be diagnosed with more than one disorder; therefore, these estimates are not mutually exclusive across diagnostic categories. Respondents reported similar estimates for bipolar disorder (80.3%), major depressive disorder (82.0%), and schizophrenia spectrum disorders (80.3%). Although the percentage of each diagnosis varied by the service provider, most (95.1%) of the sample reported that their clients were dually diagnosed with SMI and substance use disorders.

### Analytical Strategy

The data analysis process followed a systematic and iterative approach. NVivo 12 software was utilized for analyzing the data, employing thematic qualitative analysis with a single coder. The coding process took place over four phases. Initially, the sole coder created a preliminary codebook based on the review of two randomly selected interviews. Subsequently, the coder analyzed two additional interviews using the preliminary codebook. No changes to the codebook were necessary. The remaining interviews were then coded employing a hybrid coding method that incorporated both deductive and inductive approaches (Fereday & Muir-Cochrane, 2006). Prior research was utilized to develop initial codes (deductive), which were further refined based on the interviews themselves (inductive). This approach focused on the manifest content, prioritizing objectivity and reliability. Finally, given that there was only one coder, after a two-month interval the coding process was repeated for ten randomly selected interviews to allow for comparison of codes between two time points and to assess inter-rater reliability, which was found to be high, with kappa values exceeding 0.95. Additionally, throughout the coding process, analytic memos and notes were maintained for documentation (Saldana, 2015).

## Results

This study investigated the effects of telemedicine on the accessibility of mental health treatment for those with SMI and criminal justice involvement. In analyzing narratives surrounding the implementation of telemedicine in the treatment of criminal justice clients with SMI, this study considered two research questions. First, did telemedicine increase or decrease access to mental health treatment? Second, what existing or new barriers were introduced due to telemedicine. Results suggest that CMHCs saw significant reductions in resources and programming (e.g., group therapy) that were quickly replaced with telemedicine, alleviating barriers for those with transportation and childcare responsibilities while still maintaining social distancing. Increased client engagement was also cited as a positive consequence of telemedicine. However, service providers expressed concern related to clients’ reliable access to internet, the difficulties involved in maintaining confidentiality, and the limitations to the client information that can be gathered when meeting virtually. The next sections will delineate these themes.

### Reductions in CMHC resources and programming

Due to the pandemic, CMHCs were forced to indefinitely suspend in-person treatment and group programing. This was particularly problematic for interviewees like Service Providers 106 and 265 who predominantly provided treatment through group activities or who regularly offered services in county jails. As such, CMHCs quickly replaced in-person programing with alternative treatment methods such as incorporating YouTube videos and the implementation of telemedicine.

### Alleviated barriers and increased client engagement

Prior to telemedicine, interviewees regularly (*n* = 20) discussed clients’ transportation barriers, often stating that clients could not make their appointments due to a lack of reliable personal or public transportation. Many criminal justice clients may not have a valid driver’s license due to their involvement in the criminal justice system. They may also reside in rural areas where there is limited or no access to Medicaid’s medical transportation program (hereinafter referred to as Medicaid taxi cabs). Service providers described Medicaid taxi cabs as undependable and, therefore, a significant source of stress for clients. If clients consistently miss appointments, they are at risk of being labeled as non-compliant with criminal justice requirements and in turn may face periods of jail or prison incarceration. In other cases, service providers are forced to close out their non-active client’s case and the client must begin the intake process all over again, which can sometimes take months.

Telemedicine eliminated the need for reliable transportation as clients could now engage in treatment from any location of their choosing. One service provider (SP 191) referred to removed transportation barriers as a “game changer” due to the previous pervasive issues related to client transportation. Additionally, although only mentioned twice (Administrative Service Providers 120 and 177), it is significant that service providers who were responsible for transporting clients for intake evaluations and prescriptions also referred to the benefits of transitioning to telemedicine.

With the elimination of the transportation barrier, clients also experienced relief related to childcare issues. Although service providers did not mention client barriers or concerns regarding childcare responsibilities prior to the pandemic, upon the implementation of telemedicine Service Provider 265 described how attendance improved for parents:

Another great thing that I’ve seen too, a lot of these people who couldn’t go to meetings or get hooked up with services or able to because of children, having children in the home, not having a babysitter, not having family support. You know, they are now able to get online with their kids in the background and these services are being produced, which it’s kinda like…we should have been doing this a long time ago…there’s so many people that have called and said ‘I’m so sorry I’m gonna have to cancel, but I don’t have a babysitter or there’s just no way.’

Clearly, telemedicine addressed transportation and childcare barriers and allowed clients to attend their appointments more regularly, as well as to take advantage of additional evidence-based programming. Along with improvements in attendance, service providers also witnessed increased client engagement. This was notable for a population defined in the following terms by one service provider, “[i]t is hard to get them engaged and keep them engaged and to get them to come to the office for treatment” (Service Provider 120). The same service provider went on to say that before telemedicine, “if they weren’t coming in or they weren’t responding when a home base worker was trying to make an appointment, our hands were tied.”

According to participants, telemedicine was also effective in reducing barriers to accessing treatment for clients who are elderly, ill, or physically disabled. Service Provider 288 described how telemedicine can benefit this population of clients:

This whole COVID-19 thing and the fact that we have to work from home, and we have to work remotely… this has probably opened doors to being able to provide more services because now we can do this remotely. Whereas in the past, they wouldn’t let us, so, we have an elderly person that’s at home that cannot get her body into our facility. She doesn’t have transportation, her health is bad, we’ve got other people that aren’t elderly that don’t have transportation their health is bad, they cannot walk from that door to that door without gasping for breath and they want to be on the phone, they want to get their therapy, but they can’t. Because we can’t get paid for it. So, we need to use our time for what we can get paid for so we can stay open. You know if we’re not staying open then they don’t get anything anyway.

In this way, not only do virtual treatment options improve access to treatment by means of eliminating transportation barriers, but they also improve client attendance and engagement, and CMHCs also benefit in the process.

### New barriers to treatment

Although service providers generally held positive views regarding virtual treatment options, seven service providers discussed new challenges related to treating this population through telemedicine. First, service providers expressed concern with clients having internet or the financial resources to purchase internet services through their cell phones. Second, beyond internet access, confidentiality was also a significant concern. With the implementation of telemedicine, clients were able to participate virtually while service providers also worked from the comfort of their own homes. This new treatment modality coupled with the reality of families sharing living spaces brought about concerns with maintaining confidentiality. Like their clients who meet with them virtually within their homes, service providers working from home may also need to share their space with other family members who could overhear these private conversations. Female service providers with children and no childcare options outside of the home were forced to juggle their own childcare responsibilities while also providing mental health treatment to clients. Service providers could also face concerns that were similar to those of their clients who resided with partners, spouses, and children in the home and whether it was appropriate (even with the client’s permission) to continue telemedicine appointments while others were present. Take one scenario discussed by Service Provider 265 for example:

I had to cut off a Zoom meeting the other day because my husband had come home from work early. And I had to say, even though you probably couldn’t hear him in the background, ‘hey my husband is in the background. I’m gonna have to, you know I’m gonna have to exit. I’m sorry, just confidentiality and people don’t really seem to mind that. They’re ok. They’re like ‘no it’s ok.’ And I’m surprised by that, because and maybe it’s my age like I said because I’m not so sure that I would talk to my therapist over the phone. You know I would be reluctant.

Service providers also expressed a third concern related to the limitations of telemedicine appointments. Although meeting virtually provided an additional source of clinically relevant information in that providers were able to see a client’s living environment (Service Provider 265), it was more difficult to collect reliable information related to the client’s hygiene, evidence of active substance use, and their overall demeanor. Service provider 288 described this best when they stated that face-to-face appointments are preferrable because, “when you’re face to face you get energy, you get you know, you can feel a modicum of what they’re feeling when you’re with them. Now whether that’s true or not, the way we’re doing it here I can only see you from here up. I don’t see what your legs are doing. For all I know they’re shaking uncontrollably. If I saw them, I would kind of comment on them and I would have a little bit more information.”

## Discussion

The COVID-19 pandemic brought about an unprecedented disruption to mental health treatment. Consistent with prior literature, findings from the current study suggest that telemedicine played a crucial role in mitigating these disruptions and addressing delays to mental health treatment on a broad scale. Although the results of this study suggest that telemedicine was effective in addressing certain barriers that disproportionately impact criminal justice-involved individuals, findings also highlight new and unanticipated challenges that must be addressed to equitably benefit all clients. This section discusses findings related to the benefits and challenges raised by the implementation of telemedicine.

Interviews with service providers suggest that telemedicine was beneficial in providing mental health treatment to clients during a time when in-person treatment and program was impossible. Not only did telemedicine increase opportunities to continue treatment at this time, but it unintentionally addressed several existing barriers disproportionately affecting this population as well. For many criminal justice-involved individuals, transportation issues are a reoccurring concern that can be partially alleviated through telemedicine (Baldwin et al., 2020; Oluyede et al., 2022). For those in rural areas, the unreliable nature of Medicaid taxis further compounded the pre-existing issues. According to service providers, telemedicine offered access to treatment from a location of their choosing, effectively eliminating transportation barriers.

Alleviating transportation barriers, as also evidenced by prior research, simultaneously addressed issues related to childcare responsibilities (Katzow et al., 2020). Service providers explained that clients no longer had to struggle to find babysitters or family to help with childcare, as they could receive treatment without leaving their homes. In some cases, telemedicine also addressed barriers for elderly and disabled clients, who previously found it difficult to physically access treatment in person. This flexibility not only improved client attendance, but consistent with prior research also increased client engagement (Schwalbe & Koetzle, 2021; Swann et al., 2022), which has historically constituted a challenge when working with criminal justice clients. Research suggests that clients who invest in the treatment approach have better results (Rotter & Carr, 2011; Staudt et al., 2012), therefore, if telemedicine promotes engagement and satisfaction, one possible outcome may be a correlated measurable reduction in recidivism over time.

Although telemedicine alleviated several significant barriers, it also introduced unanticipated challenges for service providers. As is evident from prior research in this area, service providers expressed concerns related to their client’s reliable access to the internet (Dir et al., 2022; Krider & Parker, 2021; Schwalbe & Koetzle, 2021; Zaller et al., 2023), which is a requirement of the treatment delivered through virtual means. Financial constraints and access to the internet could prevent clients from fully engaging in virtual treatment (Song et al., 2021).

Confidentiality emerged as another concern for service providers. Telemedicine blurred the lines between personal and professional spaces, as clients and service providers participated in treatment from their homes. Prior research on client confidentiality, although limited, offer mixed results (Batastini & Morgan, 2016; Krider & Parker, 2021). Findings in this context expand our understanding of privacy concerns and highlight issues related to confidentiality beyond the client; service providers were also concerned with potential breaches to privacy with family members within earshot of both the client and service provider. However, confidentiality concerns may have lessened as the pandemic subsided and service providers transitioned back to professional spaces and family members went back to work. Finally, unexplored by earlier studies, telemedicine also presented challenges for service providers looking to gather comprehensive client information related to their hygiene, substance use, and overall demeanor.

### Limitations

Before discussing the implications of these findings on policy, practice, and future research, it is important to address several limitations that are inherent to this research. First, although the service providers interviewed here were recruited from over half of the CMHCs located in Indiana, 21 organizations did not respond or declined participation in this study. Second, there is no accessible list of service providers who work in Indiana CMHCs; therefore, sampling was limited to a combination of purposive and snowball methods. Because of this, the findings may be limited due to selection bias. These limitations limit the generalizability of this study’s findings. Additionally, this study’s original purpose was not to examine the effects of COVID-19 or telemedicine on mental health treatment delivery, so saturation of qualitative themes may not have been met. Relatedly, while this study offers valuable insights into the implementation of telemedicine, it is important to recognize that the context of the COVID-19 pandemic may limit our understanding of its broader applicability. The pandemic created unique and unprecedented conditions, such as widespread lockdowns, heightened health concerns, and rapid adaptation by service providers and clients. These conditions may not fully represent the typical environment in which telemedicine would operate post-pandemic. Consequently, the findings related to telemedicine’s effectiveness, accessibility, and acceptability might be influenced by the extraordinary circumstances of the pandemic, making it challenging to generalize the results to more stable and routine times. Further research is needed to evaluate telemedicine’s long-term viability and effectiveness outside the pandemic context, considering factors such as technological advancements, evolving policies, and societal attitudes toward virtual treatment services. Finally, the findings here represent only the perceptions of service providers and not of the clients receiving services, and therefore, may not provide a complete picture of the influence of telemedicine on mental health treatment.

### Implications for policy, practice, and research

The findings of this study offer significant implications for the utilization of telemedicine as a means of providing treatment to individuals involved with the criminal justice system in community settings. Firstly, they highlight the transformative potential of telemedicine in overcoming barriers to mental health treatment for this population. By addressing issues such as transportation concerns and the accommodation of clients with childcare responsibilities, telemedicine emerges as a valuable tool for accessing mental health services. Secondly, the results suggest a positive correlation between telemedicine utilization and client engagement. Given the challenges inherent to establishing rapport with criminal justice clients and concerns regarding the perception of service providers as extensions of the criminal justice system, these findings suggest that the flexibility afforded by telemedicine may enhance the overall effectiveness of mental health interventions for this group. Thirdly, while recognizing the benefits, it remains crucial to address the unforeseen challenges introduced by telemedicine. Concerns raised by service providers regarding internet accessibility, confidentiality, and limitations in gathering comprehensive client information pose significant considerations for both service providers and CMHCs more broadly. Understanding these challenges, in addition to promoting digital literacy, is essential for developing strategies aimed at optimizing the efficacy of telemedicine while ensuring the privacy of both clients and service providers.

Collectively, this study’s findings support the implementation and continuation of telemedicine for mental health treatment in criminal justice populations. The benefits of telemedicine extend beyond addressing pandemic-related challenges and far outweigh the existing barriers for improving access to treatment by a wider range of clients. Unfortunately, although telemedicine does hold promise as a vital component of mental health treatment, many states did not renew their telemedicine bills post-pandemic and the changes remained temporary (Health Resources & Services Administration, n.d.; Rubin, 2022; U.S. Department of Health and Human Services, 2023). Although, virtual treatment options do remain available under certain conditions, it is imperative that CMHCs strike a balance between the advantages of telemedicine and challenges related to confidentiality, financial limitations limiting clients’ ability to access virtual treatment, and the limitations to the information that can be gathered virtually. Further research on the delivery of mental health treatment through telemedicine, and its long-term feasibility, will be essential to ensure equitable and effective mental health treatment for not only criminal justice-involved clients, but all clients receiving mental health treatment.

## Conclusions

This study sought to investigate the impact of telemedicine services on a population of criminal justice-involved individuals with SMI who were receiving treatment in community-based treatment settings. The findings highlight the significant role that telemedicine played in mitigating disruptions to mental health treatment during the early stages of the pandemic. According to service providers, telemedicine effectively addressed barriers to treatment that disproportionately affect criminal justice populations, which also resulted in improved client attendance and engagement in treatment. Research on treatment barriers suggests that eliminating or reducing the impact of these obstacles, coupled with increased engagement in treatment, could lead to measurable reductions in recidivism over time. Findings also shed light on unanticipated challenges to treating criminal justice-involved clients through telemedicine. Concerns included clients’ reliable access to the internet, financial constraints, and confidentiality issues as treatment sessions took place in client and provider homes during mandatory lockdowns. Additionally, interviews suggest that service providers faced new limitations in gathering comprehensive client information virtually, which could impact the effectiveness of mental health treatment.

## Data Availability

The dataset generated and analyzed during the current study are not publicly available due identifiable information but are available from the corresponding author on reasonable request.
